# On Constitutional Inaptitude to Cow-Pox

**Published:** 1827-11

**Authors:** George Gregory

**Affiliations:** Physician to the Small-Pox and Vaccination Hospital.


THE LONDON
### Medical and Physical Journal.

No. 345, VOL. LVIII.] NOVEMBER, 1827. [No. 17, *New Series*.

For many fortunate discoveries in medicine, and for the detection of numerous errors, the world is indebted to the rapid circulation of Monthly Journals; and there never existed any work, to which the Faculty, in Europe and America, were under deeper obligations, than to the Medical and Physical Journal of London, now forming a long, but an invaluable, series.—RUSH.

### ORIGINAL PAPERS,

AND

### CASES OBTAINED FROM PUBLIC INSTITUTIONS AND OTHER AUTHENTIC SOURCES.

### VACCINATION.

*On Constitutional Inaptitude to Cow-Pox.* By GEORGE GREGORY, M.D. Physician to the SMALL-POX AND VACCINATION HOSPITAL.

IN a former Number of this Journal, (November 1826,) I offered some observations on what may be termed the surgery of vaccination, in the course of which it was remarked (page 413,) that failure in the operation does not necessarily presume error on the part of the practitioner, for that several circumstances in the system of the individual may prevent the success of the operation. The object of the present paper is to throw, if possible, some additional light on this subject,—to develop, as far as the author's experience permits, the doctrine of a constitutional inaptitude to cow-pox, —to point out how it may be at all times recognised,—and how far an acquaintance with such a principle may be turned to advantage.

In the course of my attendance on the vaccination department of the Small-Pox Hospital, I had frequent opportunities of noticing that a certain number of children passed through the disease in a manner very different from the majority. The vesicles were small, their progress slow, their surrounding areola faint, and the constitutional disturbance accompanying their maturation very trifling,—if, indeed, at all perceptible. In proportion as my acquaintance with these instances of imperfect vaccination increased, I found that such children received the first impression of the virus with great difficulty; that in many cases, out of eight or ten punctures, two only, sometimes only one, succeeded; and that one was often two or three days behind the true period. On the other hand, I learned that in those children whose arms exhibited the most perfect specimens of genuine cow-pox, all the original incisions advanced to maturity; and by degrees the important law was impressed upon my mind, that constitutional and local aptitude and inaptitude to cow-pox accompany each other, and, the mode of vaccinating being in all cases the same, that the proportion of successful to unsuccessful incisions afforded a fair criterion of the degree to which the constitution was predisposed to receive the vaccine virus, and, as we may presume also, to profit by it.

It has been a very common remark that sickly children, those labouring under inflammatory complaints or cutaneous affections of the porriginous or herpetic kind, and others in a state of marasmus, are usually unfit, and often quite incapable of receiving, the vaccine influence. This observation has been amply borne out by my experience at the Small-Pox Hospital; but it admits of some modifications. In a few instances, where the entreaties of the mother have induced me to vaccinate an unhealthy-looking infant, I have been agreeably surprised by an abundant crop of the most perfect vaccine vesicles. Much more frequently has it happened to me to notice that a constitutional inaptitude to cow-pox shall co-exist with the most healthy aspect.

A few instances will be sufficient to illustrate the principle now laid down; after which, it will be my object to point out the practical applications of which it is susceptible.

Case I.—Jane Thomas, aged one year and six months, residing No. 16, Noble-street, Spa-fields, was brought to the Small-Pox Hospital to be vaccinated, July 26th, 1827. The child is of a pale complexion, but fat. The mother states that she has frequently taken it to the doctor, on account of what she calls a "stoppage at the chest." The child's breathing is noisy, or croupy, and has been so for a considerable time past. An elder brother died of a similar affection, when nearly the same age. I vaccinated the child in seven places.

July 30th.—Only two of the seven incisions have taken. Vaccination repeated in six places in the other arm.

August 4th.—Four of the second crop have taken effect, but the papulæ are all very small and backward.

August 6th, (twelfth day of original vaccination,)—Areola has appeared on both arms, but very faint. There has been much itching of the part, and the vesicles appear as if rubbed. They are small, and, when closely inspected, appear very different from the genuine vaccine vesicles of this period. I explained to the mother, that the child, either from its general state of health, or from some other less obvious cause, was incapable of receiving the full influence of vaccination. I told her to place little or no security upon this vaccination, but to have it repeated hereafter, perhaps in the course of a year or two, when the child's breathing was improved, and its constitution mended.

Considering that in this case vesicles had been produced, with a certain portion of surrounding inflammation, a practitioner not fully conversant with the different aspects of the disease might here have been easily deceived, and have unguardedly pronounced this vaccination as satisfactory. That such opinions were once entertained I can have no doubt, from the answers I am constantly receiving from those who enter the hospital labouring under small-pox subsequent to vaccination.

Case II.—Jane Mackey, aged two years and two months, residing No. 31, Aylesbury-street, Clerkenwell, was brought to the Small-Pox Hospital to be vaccinated, May 28, 1827. The mother informs me that, at two different periods in 1826, she had taken the child to Rowland Hill's Chapel, where vaccination is carefully performed, but that at each time the pimples died away without coming to a head. The child at the present period is apparently in perfect health. I vaccinated it carefully in fourteen places.

June 4th, (eighth day.)—Only three have taken, and these are very backward and imperfect.

The mother does not appear to have brought the child again.

Case III.—Stephen Ricketts, aged four months and a fortnight. residing No. 46, Gee-street, Goswell-street, was brought to the Small-Pox Hospital for vaccination, August 20, 1827. The child appears pale, but otherwise healthy. Nine incisions were made.

August 27th.—Only three incisions have taken, and the papulæ are small.

August 31st, (eleventh day of vaccination.)—The three vesicles are small. A slight degree of areola has surrounded them, which is however now faded. The child has never suffered, in the slightest degree, in its general health. I told the mother that she could not rely on this vaccination, and advised a repetition of the operation in the course of another year.

Case IV.—Frederick Heutsch, aged two months and a fortnight, residing No. 18, Cornwall-road, Stamford-street, was vaccinated at the Small-Pox Hospital, August 20, 1827. The child was pale, but otherwise healthy. Nine incisions made, which totally failed.

August 29th.—Vaccination repeated in the same number of places.

Sept. 7th, (tenth day of second vaccination.)—Only one of the incisions of the second vaccination have taken, the areola surrounding which is small and imperfect. The mother was informed that she was not to place reliance upon this vaccination for the permanent security of the child.

It may perhaps be demanded, in the first instance, on what authority I venture to assert that such vaccinations as have been just described are incapable of saturating the system, and of giving to the child that full measure of protection of which vaccination is certainly capable. It is true that I have, in no one instance, inoculated the child subsequently, so as to settle the question definitively; but it is unreasonable to attribute the same effects to appearances so widely different as those of perfect and of imperfect vaccination. Besides, as I have already hinted, the accounts of the prior vaccination which I receive within the walls of the hospital from those who have the misfortune to become in after-life the subjects of small-pox, together with the character of the scars on their arms, correspond, in a great number of cases, with the appearances which I have now described.

Another question, too, very naturally arises from the perusal of the foregoing statement: Are there any persons *wholly* unsusceptible of the cow-pox? And is there any reason to believe that such constitutional inaptitude to cow-pox continues through life? These are questions which, in the present state of our knowledge concerning cow-pox, it is scarcely possible to answer. Mr. Cates, of Argyll-street, has favoured me with the history of a child who has hitherto resisted every effort which has been made to imbue him with the vaccine influence; and I have met with several instances at the Small-Pox Hospital of children ineffectually vaccinated at other institutions, who continued there to resist the impression of the vaccine virus. Early in the month of June last, attempts were made to vaccinate six or eight natives of the South-Sea Islands, recently arrived in this country, and at that time living on-board the Grampus hospital-ship. They were all suffering under inflammation of the bronchial passages, and in no instance did the vaccination succeed.

A third question connected with this investigation is important with reference to practice. Does the constitutional inaptitude to cow-pox accompany that to small-pox? That is, in other words, may we reasonably presume that a child who has altogether resisted, or only imperfectly received, the vaccine influence is, for a time at least, equally unsusceptible of the small-pox? It would require a very extended sphere of observation to give a definitive answer to this question. As far as my own very limited inquiries go, (and which I acknowledge to be quite unequal to the determination of so important a point,) I should be inclined to say that the predisposition to the two complaints was the same, and that a reasonable presumption exists, that a child constitutionally inapt to cow-pox is, for the time, equally indisposed to small-pox.

That the doctrine of constitutional inaptitude to cow-pox, either complete or partial, is capable of explaining a very considerable number of the cases which we are now almost daily called upon to witness, of small-pox succeeding vaccination, I cannot entertain a doubt. That there may be other co-operating causes I can well believe; but I am daily becoming more and more convinced that, in the early years of vaccination, appearances on the arm were deemed satisfactory which had no real claim to confidence, and I fear that, even at the present moment, practitioners are too easily induced to pronounce upon the perfection of the process.

I shall add but one other reflection, which has often occurred to me in considering this subject. Many authors have indulged the notion, and among others Sir Gilbert Blane, in his Essay on Vaccination, (first published in the tenth volume of the Medico-Chirurgical Transactions,) that "if mankind were sufficiently wise and decided to vaccinate at one moment the whole of the human species who were then unprotected, small-pox would be instantaneously and for ever banished from the face of the earth." This notion proceeds on the assumption that the whole human species are constitutionally susceptible of the cow-pox, which is certainly not the case,—at least at any one given period of time. What is the proportion existing between those who are and those who are not predisposed to receive cow-pox, is a question worthy perhaps of some inquiry, but it is, of course, difficult to arrive at a satisfactory conclusion. Out of the eighty or ninety persons who, upon an average, are vaccinated weekly at the Small-Pox Hospital, I usually find three, or perhaps four, who appear incapable of benefiting by it; and there are perhaps several more whose constitutions are fitted to receive only a certain portion of good from it. If any judgment, at least, can be formed from the various appearances of the arm, and the degree of constitutional disturbance present in the several cases, (and I know not from what other sources information can be gained,) it is unreasonable to suppose that all children can be equally and uniformly affected.

What were the peculiar opinions entertained by Dr. JENNER on the subject of a constitutional inaptitude to cow-pox, I have in vain endeavoured to ascertain. Perhaps some of the readers of this Journal, either by more extensive acquaintance with his works or by personal communication with Dr. Jenner, may be able to instruct me on this head. In doing so, through the medium of this Journal, I may venture to add that they will be conferring a favour on the profession at large.

*September*, 1827.

